# Serum potassium level is associated with serum neurofilament light chain in American adult population: a cross-sectional analysis of the 2013–2014 National Health and Nutrition Examination Survey

**DOI:** 10.3389/fnagi.2025.1511881

**Published:** 2025-03-18

**Authors:** Yingming Kong, Yanghao Tai, Bin Chen, Chunzheng Li, Hao Chen, Liang Shi

**Affiliations:** ^1^Third Hospital of Shanxi Medical University, Shanxi Bethune Hospital, Shanxi Academy of Medical Sciences Tongji Shanxi Hospital, Taiyuan, China; ^2^Basic Medical College, Shanxi Medical University, Taiyuan, China; ^3^Department of Neurology, Shanxi Bethune Hospital, Shanxi Academy of Medical Science, Tongji Shanxi Hospital, Third Hospital of Shanxi Medical University, Taiyuan, China

**Keywords:** serum potassium, serum neurofilament light chain, sNfL, NHANES, population-based studies, axonal degeneration

## Abstract

**Objective:**

Serum neurofilament light chain (sNfL) is one of the most sensitive diagnostic biomarkers for a variety of neurodegenerative pathologies. Potassium, an essential electrolyte, plays a critical role in maintaining neuronal health and the proper functioning of the central nervous system (CNS). The aim of our research was to investigate the association between serum potassium level and sNfL levels.

**Methods:**

Based on the National Health and Nutrition Examination Survey (NHANES) database, we analyzed data from the 2013 to 2014 NHANES. Serum potassium concentrations were measured via ion-selective electrode (ISE) technology. The levels of sNfL were measured using a sensitive immunoassay developed by Siemens Healthineers. Our researcher analyzed the association between potassium level in serum and sNfL in American persons using multivariate logistic regression analysis and smoothed curve fitting. The consistency of these results was examined in various population subgroups.

**Results:**

A total of 1,670 participants were involved in our research, including 872 women (50.5%) and 798 men (49.5%). The median serum potassium concentration was 4.0 mmol/L and the median sNfL was 12.3 pg/ml. After adjusting for potential confounders in the full model, individuals with higher serum potassium concentrations had higher sNfL levels (Q3 vs. Q1, β = 2.86, 95% CI: 0.33–5.39, *P* = 0.027). There was a non-linear positive dose-response association between higher serum potassium concentrations and sNfL levels (*P* for non-linearity = 0.028). Based on the results of stratified analysis, the relationship was stronger in the low- and middle-income group, non-drinking and non-physical activity participants, and participants with hypertension and diabetes.

**Interpretation:**

In the cohort of American adults, a greater serum potassium concentration was linked to a higher sNfL. However, causality still needs to be further verified.

## 1 Introduction

Neurofilament proteins constitute the cytoskeleton of mature neurons (Gafson et al., 2020). Neurofilament light chain is one of its major subunits and constitutes a scaffolding protein for the neuronal cytoskeleton through spontaneous assembly. Within the central nervous system (CNS) and peripheral nerve system, serum neurofilament light chain (sNfL) is crucial in terms of conduction velocity and axon caliber (Gaetani et al., 2019). The sNfL is released into serum and cerebrospinal fluid following axonal injury or degeneration. Due to its high solubility and low molecular weight physicochemical properties, it can be detected at relatively low levels (Abu-Rumeileh et al., 2023). However, given the high risk associated with lumbar puncture for obtaining cerebrospinal fluid, the advantages of serum sNfL assays are more pronounced, including being minimally invasive and inexpensive (Tortosa-Carreres et al., 2024). As evidenced by earlier research, sNfL is thought to be a sensitive diagnostic biomarker for a variety of neurologic and neurodegenerative diseases. Previous studies have demonstrated that sNfL is thought to be a sensitive diagnostic biomarker for a variety of neurodegenerative diseases, especially for various neurological disorders characterized by axonal degeneration (Arslan and Zetterberg, 2023). Clinically, sNfL is an important biomarker for monitoring disease recurrence as well as evaluating the efficacy and prognosis of treatment, including motor neuron disease, Parkinson’s disease (PD), Alzheimer’s disease (AD), multiple sclerosis, primary psychiatric diseases, Guillain–Barre syndrome, traumatic brain injury, stroke, genetic peripheral neuropathy, and other neurological disorders (Aamodt et al., 2021; Abdelhak et al., 2023; Benkert et al., 2022; Brousse et al., 2023; Chen et al., 2022; Hwang et al., 2023; Rossor and Reilly, 2022; Teunissen et al., 2022; van Tilburg et al., 2024; Wang et al., 2024). The sNfL is therefore widely used to assess neuroinflammatory and degenerative diseases. Notably, sNfL levels are often influenced by external environmental factors (Bi et al., 2024). Notably, NfL levels are not only influenced by factors within the nervous system, but may also be regulated by external environmental factors, including the homeostasis of metal ions. These ions play critical roles in the nervous system and are involved in a variety of biochemical processes such as cell signaling, enzymatic reactions, and maintenance of neurotransmitter homeostasis (Cicero et al., 2017; Lai et al., 2024). The delicate balance of metal ions is critical for neuronal function. Therefore, the impact of metal ions cannot be ignored when assessing the diagnostic significance of NfL in neurodegenerative diseases.

Metal ions are ubiquitously present in the CNS and play an important role in maintaining neurophysiological homeostasis and neuronal function. Specifically, metal ions can function as receptors, transporter proteins, enzymes, transcription factors, and so on. Imbalances in ion concentrations can affect the physiological activities of nerve cells, even leading to cell death and affecting the health of the organism (Kawabata, 2022). Therefore, metal homeostasis is important for the maintenance of neurophysiological homeostasis. Emerging studies have shown that various neurodegenerative diseases are associated with metal ion imbalance, such as AD, vascular dementia, PD, and amyotrophic lateral sclerosis (Bisaglia and Bubacco, 2020; Carri et al., 2003; Tabet et al., 2001; Wang et al., 2020). For example, it has been observed in established experimental animal models that various metal ions can synergistically induce neurotoxic effects, which in turn lead to the development of vascular dementia (Tanaka and Kawahara, 2017). In the brain, the concentration of metal ions is usually higher than in other parts of the body. Cells have evolved a variety of metal regulatory mechanisms to maintain homeostasis in the intra- and extracellular environments, especially in the CNS (Jomova et al., 2022). Once the homeostatic balance of metals in the CNS is disrupted, progressive neurodegeneration, neuronal loss, protein deposition, and a variety of oxidative stress and inflammatory responses occur (Liu et al., 2022). For the dynamic balance of metal ions, blood potassium plays an important role.

It has been well established that sNfL is associated with a variety of neurodegenerative diseases (Gao et al., 2023). Based on the common outcome of neurodegenerative diseases and the research evidence that elevated potassium levels lead to myelin destruction (Boerio et al., 2014), our study aimed to elucidate the relationship between serum potassium ions and sNfL from a cross-sectional perspective using a large sample of data from the National Health and Nutrition Examination Survey (NHANES).

## 2 Materials and methods

### 2.1 Data sources

The 2013–2014 NHANES datasets were the original information resource for the present analysis. The NHANES database provides a comprehensive range on each participant’s laboratory tests, disease information, human examinations, and population characteristics. The NHANES database ensures data reliability through nationally representative sampling and standardized protocols, the use of the most advanced and reliable techniques of information collection and organizational and statistical methods for data analysis. This data is subsequently subjected to additional analysis and utilized to study risk factors for a variety of diseases. A sophisticated multi-stage probabilistic approach was used in this study to ensure that the samples used are accurate and representative. Each participant signed up an informed agreement and the qualification of human subjects in NHANES was approachable via the National Center for Health Statistics (NCHS) Ethics Review Board.

### 2.2 Research population

**FIGURE 1:**
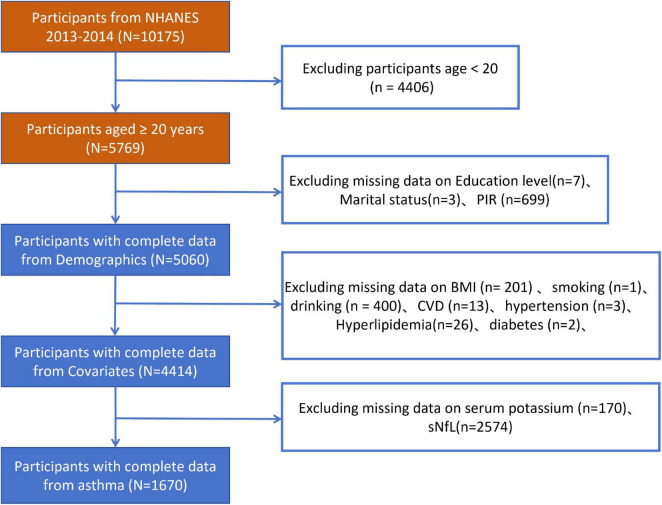
Flowchart depicting participant selection in the study.

In this research, a total of 10,175 individuals were chosen from the NHANES 2013–2014 dataset, as depicted in Figure 1. Exclusions were made for participants with incomplete age data under 20 years (*n* = 4,406), missing educational information (*n* = 7), unknown marital status (*n* = 3), and lacking economic data (*n* = 699). Additionally, subjects with incomplete data on various factors such as BMI (*n* = 201), smoking habits (*n* = 1), alcohol consumption (*n* = 400), CVD (*n* = 13), hypertension (*n* = 3), hyperlipidemia (*n* = 26), and diabetes (*n* = 2) were also ruled out. The study’s criteria necessitated a dataset that included both serum potassium and sNfL levels; hence, those without serum potassium data (*n* = 170) and those without sNfL data (*n* = 2,574) were not considered. Consequently, the final analysis comprised 1,670 participants (Figure 1).

### 2.3 Definition of exposed variables and outcome variable

The measurement step of sNfL levels was performed on the high-throughput fully automated immunoassay platform Atellica, based on a highly sensitive immunoassay developed by Siemens Healthineers. In the NHANES database for the period 2013–2014, participants aged 20–75 years were included, and their serum samples were measured and processed at mobile examination centers. These participants agreed to preserve their blood samples for further study analysis, while their original serum samples were also securely stored. For more information and detailed agreements, please visit the following link: http://wwwn.cdc.gov/Nchs/Nhanes/2013-2014/SSSNFL_H.htm. The minimum limit of quantification (LLOQ) for this assay was set at 3.9 pg/ml, which was derived from the analysis of replicate test results from 44 low-concentration neurofilament protein light chain (NfL) samples. The criterion for determining LLOQ is that the coefficient of variation (CV) does not exceed 20%. For analytes that measure below LLOQ, the result field should be populated with an imputation value equal to LLOQ divided by root number 2. For analytes that measure more than ULOQ, the result field should be populated with a value equal to ULOQ multiplied by the root number 2. The concentration of potassium is determined by measuring the ionic activity in the solution. The DxC800 instrument uses ion-selective electrode (ISE) technology to determine potassium levels in biological fluid samples, either indirectly or diluted. The potassium electrode used by the device contains a valinomycin membrane that is able to sense potassium ions and produce a potential change that follows the Nernst equation, which allows the concentration of potassium in solution to be calculated.

### 2.4 Covariates

Covariates in this investigation were variables that might affect the relationship between the levels of serum potassium and sNfL. Covariates considered in this study included sociodemographic factors including age (≤40, 41–60, >60), educational level (high school graduate/GED or equivalent, college graduate or above, less than 12th grade), ethnicity (other Hispanic, other race, non-Hispanic black, non-Hispanic white, and Mexican American), family poverty income ratio (PIR) (≤1.0, 1.1–4.0, >4.0), and marital condition (living alone, married and living with partner). The researchers also included health related factors, hypertension, diabetes, alcohol consumption, physical activity, smoking status, hyperlipidemia, and cardiovascular diseases (all described as yes/no). PIR is the measurement of socioeconomic position, defined as the ratio of income to the United States Census Bureau’s poverty level for a family. Individuals are classified as present smokers, never smokers, and former smokers based on “Do you personally use cigarette or tobacco?” and “Smoked more than 100 cigarettes in your lifetime.” Based on “Drink at least twelve times in a lifetime or a year,” the definition of a drinker includes current, never, and former drinkers. According to questionnaire information, self-reported disease was also considered, including diabetes. The diagnosed of cardiovascular disease, hypertension, and hyperlipidemia was regarded as the basis of a self-reported history of specific diseases. The definition of cardiovascular disease includes coronary heart disease, stroke, congestive heart failure, angina, and heart attack. Laboratory data include alanine aminotransferase, aspartate aminotransferase, alkaline phosphatase, total calcium, chloride, uric acid, direct HDL-cholesterol, triglyceride, LDL-cholesterol, and total cholesterol.

### 2.5 Statistical methods

Continuous and categorical variables were used to characterize participants at baseline. Continuous ones are described in terms of mean ± standard deviation and categorical variables are described by numbers and percentages. Weighted *t*-tests and Chi-square tests were conducted for comparisons of baseline characteristics among these participants. By assessing the corrected OR and 95% CI, the study conducted the weighted linear regression model, to investigate the connection between serum potassium level and sNfL among patients. Three models were used by us with varying degrees of modification for covariates (model 1, unadjusted for covariates; model 2, modified for age, gender, race, education level, marital status, and PIR; and model 3, with adjustments for age, gender, race, education level, marital status, PIR, smoking status, alcohol use, hypertension, diabetes, cardiovascular disease, and laboratory test indicators. In all three models, continuous variable serum potassium was used by our researchers. In addition, dose-response association between serum potassium and sNfL among patients assessed by using restricted cubic spline (RCS). The multiple linear regression models included the continuous and categorical models. The serum potassium was divided into tertiles, and then the linear trends were conducted via considering the median value of every subgroup as the continuous variable. In addition, our researchers conducted the subgroup analyses by general information, disease condition, alcohol consumption, smoking status, physical activity, and interaction analyses were performed to examine whether there were different associations between subgroups. R 4.3.3 and SPSS 26.0 were used for all statistical analyses carried out in this investigation. In terms of statistics, a bilateral *P*-value of less than 0.05 was deemed significant.

## 3 Result

### 3.1 Baseline characteristics of participants

This study recruited 10,175 individuals from NHANES 2013–2014. The flowchart of participant inclusion and exclusion is shown in Figure 1. People with incomplete information on age <20 years, sociodemographic data, covariates were excluded, and the remaining 4,414 participants were retained. In addition, after excluding participants who had incomplete data on serum potassium, the remaining 4,244 participants were considered in our investigation. After excluding incomplete data on sNfL, the information of the remaining 1,670 participants was retained. The characteristics of participants involved in this investigation at baseline were presented in Table 1. A total of 872 women (50.5%) and 798 men (49.5%) were included in the study. Significant differences were observed between males and females in marital status, smoking, alcohol consumption, hypertension, intense physical exercise, body mass index, aspartate aminotransferase levels (SAS), alanine aminotransferase levels (SAT), total calcium levels (CA), cholesterol levels (CH), chloride levels (CL), uric acid levels (UA), high-density lipoprotein levels, total cholesterol levels (TC), and triglyceride levels (TG). The median serum potassium concentration was 4.0 mmol/L and the median sNfL was 12.3 pg/ml.

**TABLE 1:** Baseline characteristics of participants based on sNfL.

Characteristics	*N*	Participant no. (weighted, %)		*P*-value
		**Female**	**Male**	
*N*	1,670	872 (50.5%)	798 (49.5%)	
Age				0.650
20–40	632	322 (41.0%)	310 (41.8%)	
41–60	639	335 (38.1%)	304 (39.0%)	
61–80	399	215 (20.9%)	184 (19.1%)	
Race				0.785
Mexican American	233	119 (8.7%)	104 (9.1%)	
Other Hispanic	154	87 (5.7%)	67 (5.3%)	
Non-Hispanic White	770	393 (65.7%)	377 (69.1%)	
Non-Hispanic Black	290	150 (12.3%)	140 (9.8%)	
Other race – including multi-racial	233	123 (7.6%)	110 (6.7%)	
Education level				0.065
Less than 12th grade	322	158 (13.4%)	164 (14.8%)	
High school graduate/GED or equivalent	347	168 (18.5%)	179 (21.7%)	
College graduate or above	1,001	546 (68.1%)	455 (63.5%)	
Marital status				0.001
Married and living with partner	1,030	506 (62.4%)	524 (67.9%)	
Living alone	640	366 (37.6%)	274 (32.1%)	
PIR				0.09
≤1.0	405	223 (19.5%)	182 (15.9%)	
1.1–4.0	800	425 (45.7%)	375 (45.1%)	
>4.0	465	224 (34.9%)	241 (39.0%)	
Smoking				<0.001
Never	935	551 (62.4%)	384 (50.4%)	
Past	379	148 (17.2%)	231 (29.1%)	
Now	356	173 (20.4%)	183 (20.4%)	
Drinking				<0.001
Never	202	143 (13.1%)	59 (7.5%)	
Past	218	155 (14.9%)	63 (5.7%)	
Now	1,250	574 (72.0%)	676 (86.8%)	
Diabetes				0.651
Yes	186	96 (9.2%)	90 (8.7%)	
No	1,436	754 (88.0%)	682 (88.6%)	
Pre-diabetes	48	22 (2.8%)	26 (2.7%)	
Hypertension				0.008
Yes	586	332 (35.8%)	254 (29.0%)	
No	1,084	540 (64.2%)	544 (71.0%)	
Hypercholesterolemia				0.408
Yes	567	288 (32.5%)	279 (30.4%)	
No	1,103	584 (67.5%)	519 (65.7%)	
CVD				0.287
Yes	138	66 (6.3%)	72 (8.1%)	
No	1,532	806 (93.7%)	726 (91.9%)	
Vigorous recreational activities				<0.001
Yes	418	186 (22.6%)	232 (30.5%)	
No	1,252	686 (77.4%)	566 (69.5%)	
Moderate recreational activities				0.06
Yes	708	389 (44.5%)	319 (41.5%)	
No	962	483 (55.5%)	479 (58.5%)	
BMI	1,670	29.83 ± 8.20 (29.92 ± 8.25)	28.59 ± 6.38 (28.72 ± 6.44)	0.001
ALP	1,670	65.62 ± 22.92 (64.17 ± 22.44)	64.68 ± 20.64 (63.33 ± 22.20)	0.377
AST	1,670	23.09 ± 15.69 (23.49 ± 19.68)	87.61 ± 33.78 (27.02 ± 26.43)	<0.001
ALT	1,670	21.11 ± 14.88 (21.57 ± 16.73)	29.08 ± 15.51 (28.84 ± 17.72)	<0.001
CA	1,670	2.35 ± 0.10 (2.35 ± 0.11)	2.36 ± 0.08 (2.36 ± 0.08)	<0.001
CH	1,670	5.04 ± 1.09 (5.04 ± 1.09)	4.83 ± 1.04 (4.85 ± 1.02)	<0.001
CL	1,670	104.59 ± 2.68 (104.69 ± 2.63)	104.04 ± 2.54 (104.05 ± 2.53)	<0.001
UA	1,670	287.82 ± 71.95 (285.37 ± 70.93)	363.93 ± 76.94 (365.01 ± 76.12)	<0.001
HDL	1,670	1.53 ± 0.42 (1.54 ± 0.42)	1.24 ± 0.34 (1.25 ± 0.35)	<0.001
TC	1,670	4.98 ± 1.06 (4.98 ± 1.06)	4.79 ± 1.34 (4.82 ± 1.03)	<0.001
TG	1,670	1.28 ± 1.78 (1.24 ± 1.56)	1.49 ± 1.25 (1.47 ± 1.24)	0.006
LDL	1,670	2.88 ± 0.88 (2.89 ± 0.89)	2.90 ± 0.89 (2.92 ± 0.88)	0.728

Continuous variables were presented as mean with standard deviation (mean ± SD), and categorical variables were expressed as proportion. Continuous variables were analyzed via one-way ANOVA; categorical variables were analyzed using the Chi-square test or the Fisher’s exact test, and *P*-value < 0.05 was considered statistically significant.

### 3.2 Association between serum potassium and sNfL

This crude model showed a significant association between serum potassium levels and sNfL levels, with a 6.87 increase in sNfL for each unit increase in serum potassium (β = 6.87, 95% CI: 4.05–9.68, *P* < 0.001). Model 2 adjusted for sociodemographic factors (marital status, poverty rate, race, age, sex, and education level) and showed a 4.87 increase in sNfL (β = 4.87, 95% CI: 2.09–7.66, *P* < 0.001). Model 3 was further adjusted from model 2 for BMI, smoking status, alcohol use, cardiovascular disease, vigorous recreational activities, moderate recreational activities, hypertension, hyperlipidemia, diabetes and laboratory test indicators as covariates, and the results showed a significant increase in sNfL of 5.15 per unit increase in serum potassium (β = 5.15, 95% CI: 2.25–8.04, *P* < 0.001).

**FIGURE 2:**
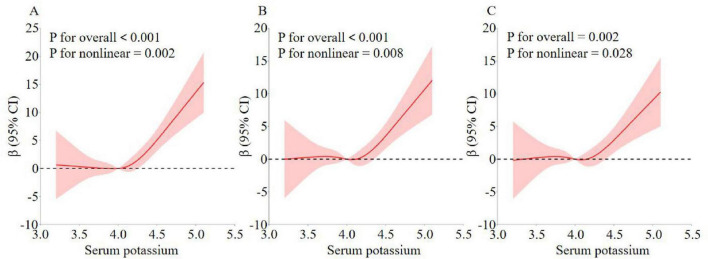
Analysis of the measured response relationship between potassium and sNfL. **(A)** Model 1 was adjusted for none. **(B)** Model 2 was adjusted for age, gender, race, education level, marital status, and PIR. **(C)** Model 3 was adjusted for age, gender, race, education level, marital status, PIR, BMI, smoking status, alcohol use, cardiovascular disease, vigorous recreational activities, moderate recreational activities, hypertension, hyperlipidemia, diabetes, and laboratory test indicators. The solid red line indicates the OR and the red shaded area indicates the 95% CI.

As shown in Table 2, serum potassium was also categorized in three equal parts and compared with the first part used as a reference. In all three models, there was a significant dose-response trend with increasing serum potassium (*P* < 0.05 for trend). Specifically, in model 1, sNfL increased by 4.36 units in the Q3 group compared with the Q1 group (β = 4.36, 95% CI: 1.86–6.85, *P* < 0.001). In model 2, sNfL increased by 2.87 units in group Q3 compared with group Q1 (β = 2.87, 95% CI: 0.41–5.33, *P* = 0.021). In model 3, sNfL increased by 2.86 units in group Q3 compared with group Q1 (β = 2.86, 95% CI: 0.33–5.39, *P* = 0.027). Although the increasing trend in model 3 was reduced compared to models 1 and 2, the association was still statistically significant. In addition, in Figure 2, the non-linear relationship between serum potassium and sNfL was explored using RCS curve regression. Figure 2 shows the results of multiple linear regression using RCS, revealing linear and positive correlations between serum potassium and sNfL levels in models 1, 2, and 3 (model 1 *P* for non-linearity = 0.002, model 2 *P* for non-linearity = 0.008, and model 3 *P* for non-linearity = 0.028).

**TABLE 2:** Weighted regression models and trend tests elucidating the association between potassium and sNfL.

Potassium	sNfL β (95% CI)					
	**Model 1**	***P*-value**	**Model 2**	***P*-value**	**Model 3**	***P*-value**
Continuous	6.87 (4.05–9.68)	<0.001	4.87 (2.09–7.66)	<0.001	5.15 (2.25–8.04)	<0.001
**Potassium tertiles**						
Q1	0.00 (Reference)		0.00 (Reference)		0.00 (Reference)	
Q2	1.49 (−0.95 to 3.92)	0.231	1.09 (−1.27 to 3.44)	0.365	1.90 (−0.47 to 4.27)	0.116
Q3	4.36 (1.86–6.85)	<0.001	2.87 (0.41–5.33)	0.023	2.86 (0.33–5.39)	0.027
*P* for trend		<0.001		0.021		0.028

Model 1 was adjusted for none. Model 2 was adjusted for age, gender, race, education level, marital status, and PIR. Model 3 was adjusted for age, gender, race, education level, marital status, PIR, BMI, smoking status, alcohol use, cardiovascular disease, vigorous recreational activities, moderate recreational activities, hypertension, hyperlipidemia, diabetes, and laboratory test indicators.

### 3.3 Subgroup analysis

To further explore the factors influencing the association between serum potassium and sNfL, we executed a stratified analysis, factoring in a spectrum of sociodemographic determinants(gender, age, ethnicity, educational attainment, marital status, and economic disparity), smoking, alcohol consumption, physical activity, and disease status (hypertension, hyperlipidemia, diabetes, and CVD). The nuanced relationships, elucidated through weighted logistic regression and detailed in Table 3, indicated a lack of significant interaction between serum potassium and sNfL across gender (*P* = 0.325), age (*P* = 0.957), education (*P* = 0.467), marital status (*P* = 0.950), smoking (*P* = 0.731), cardiovascular disease (*P* = 0.110), and hyperlipidemia (*P* = 0.223) strata.

**TABLE 3:** Subgroup analysis of the association between potassium and sNfl.

Subgroup	OR (95% CI)	*P*-value	*P* for interaction
Gender			0.325
Male	8.13 (4.28 ∼ 11.98)	<0.001	
Female	5.27 (1.10 ∼ 9.44)	0.013	
Age			0.957
20–40	4.69 (1.47 ∼ 7.91)	0.004	
41–60	5.61 (1.52 ∼ 9.70)	0.007	
61–80	5.53 (−1.83 ∼ 12.89)	0.141	
Race			0.011
Mexican American	9.26 (3.74 ∼ 14.77)	0.001	
Other Hispanic	4.03 (−0.70 ∼ 8.77)	0.097	
Non-Hispanic White	4.02 (−0.72 ∼ 8.77)	0.097	
Non-Hispanic Black	4.58 (−0.95 ∼ 10.12)	0.106	
Other race – including multi-racial	20.89 (12.11 ∼ 29.67)	<0.001	
Education level			0.467
Less than 12th grade	9.76 (4.91 ∼ 14.62)	<0.001	
High school graduate/GED or equivalent	4.27 (−0.67 ∼ 9.21)	0.091	
College graduate or above	6.87 (2.76 ∼ 10.99)	0.001	
Marital status			0.950
Married and living with partner	6.78 (2.70 ∼ 10.86)	0.001	
Living alone	6.97 (3.49 ∼ 10.45)	<0.001	
PIR			0.022
≤1.0	11.48 (5.60 ∼ 17.36)	<0.001	
1.1–4.0	7.62 (3.11 ∼ 12.13)	<0.001	
>4.0	0.58 (−3.09 ∼ 4.25)	0.756	
CVD			0.110
No	6.73 (4.23 ∼ 9.22)	<0.001	
Yes	0.11 (−16.02 ∼ 16.24)	0.989	
Vigorous recreational activities			0.007
Yes	0.37 (−2.63 ∼ 3.37)	0.807	
No	9.26 (5.66 ∼ 12.86)	<0.001	
Moderate recreational activities			0.039
Yes	3.02 (−1.89 ∼ 7.93)	0.228	
No	9.13 (5.78 ∼ 12.49)	<0.001	
Hypertension			0.004
Yes	11.20 (5.03 ∼ 17.38)	<0.001	
No	3.06 (0.93 ∼ 5.19)	0.005	
Hypercholesterolemia			0.233
Yes	8.50 (2.51 ∼ 14.50)	0.006	
No	5.03 (2.31 ∼ 7.75)	<0.001	
Smoking			0.731
Never	7.45 (4.24 ∼ 10.66)	<0.001	
Past	4.96 (−3.02 ∼ 12.94)	0.224	
Now	5.46 (0.43 ∼ 10.50)	0.034	
Drinking			<0.001
Never	28.53 (17.92 ∼ 39.14)	<0.001	
Past	9.33 (3.36 ∼ 15.30)	0.002	
Now	3.56 (−0.39 ∼ 6.74)	0.028	
Diabetes			<0.001
Yes	25.19 (13.44 ∼ 36.94)	<0.001	
No	2.40 (0.39 ∼ 5.18)	0.092	
Pre-diabetes	7.04 (0.29 ∼ 13.79)	0.047	

Within the ethnic groups, robust positive correlations between serum potassium and sNfL were exclusively noted among Mexican American individuals (β = 9.26, 95% CI: 3.74–14.77, *P* = 0.001) and those of other ethnicities (β = 20.89, 95% CI: 12.11–29.67, *P* < 0.001). Economic status, as gauged by the poverty-to-income ratio (PIR), revealed significant positive associations within the low-income (β = 11.48, 95% CI: 5.60–17.36, *P* < 0.001) and middle-income (β = 7.62, 95% CI: 3.11–12.13, *P* < 0.001) cohorts. In the subgroup of alcohol consumption, the most pronounced positive correlation was detected among those who had never consumed alcohol (β = 28.53, 95% CI: 17.92–39.14, *P* < 0.001). In the physical activity subgroup, a significant positive correlation between serum potassium and sNfL was observed in participants without vigorous or moderate physical activity (β = 9.26, 95% CI: 5.66–12.86, *P* < 0.001; β = 9.13, 95% CI: 5.78–12.49, *P* < 0.001). Furthermore, pronounced positive associations were identified among participants with hypertension (β = 11.20, 95% CI: 5.03–17.38, *P* < 0.001) and diabetes (β = 25.19, 95% CI: 13.44–36.94, *P* < 0.001).

## 4 Discussion

Our investigation primarily examined the correlation between the serum potassium and sNfL among the American adults using the NHANES database. This research involved in a sample size of 1,670 individuals. Weighted multifactorial liner regression analysis revealed the positive and statistically significant association observed between serum potassium and sNfL in American adult individuals after all covariates with control. Besides, RCS curves revealed a non-linear positive association between the serum potassium and sNfL the American adult individuals. Furthermore, the level of sNfL increased progressively with increasing serum potassium tertiles. Subgroup analyses revealed no significant interaction effects among gender, age, education, marital status, smoking status, cardiovascular disease, hyperlipidemia, but the statistical relationship between serum potassium and sNfL remained consistent. However, the association was affected in specific ethnic groups (Mexican Americans and other races), low-to-middle income participants, those who had never consumed alcohol and were inactive, and participants with hypertension and diabetes.

Physical activity transiently alters serum potassium levels via intracellular shifts during muscle contraction and renal excretion, as well as influencing potassium excretion through urinary discharge and sweating, resulting in a non-significant increase in sNfL levels in physically active people. Patients with diabetes mellitus, especially those with chronic kidney disease (CKD), are prone to disorders of potassium metabolism, especially hyperkalemia due to the progression of renal disease or the use of renin-angiotensin-aldosterone blockers, and also in diabetic patients themselves in the hyperglycemic state, where the polyol pathway is activated, leading to intracellular accumulation of sorbitol, which in turn leads to peripheral neuropathy and the consequent elevation of serum sNfL. Based on the common background of hyperkalemia and nerve injury in diabetic patients, the problem of nerve injury in patients with diabetes mellitus combined with hyperkalemia deserves more attention from us in the future. In hypertensive patients, elevated blood pressure may impair renal function and affect potassium excretion, thus leading to elevated serum potassium, so the relationship between elevated serum potassium and sNfL is more pronounced in the hypertensive population. Alcohol consumption usually leads to electrolyte disorders, and people who have consumed alcohol usually have poor dietary habits that affect potassium balance, resulting in a less significant association between elevated serum potassium and sNfL in this population. Studies have shown that smoking may indirectly affect kidney function (Mortensen et al., 2011), such as a decrease in glomerular filtration rate or a decrease in the ability of the tubules to secrete potassium, leading to an increase in the excretion of potassium ions from the body, which further reduces the potassium level in the blood. Smoking may therefore be a risk factor for hyperkalemia, and it remains inconclusive whether it ultimately leads to an increase or decrease in neurofilament chain protein. Meanwhile, it has been demonstrated that elevated serum potassium levels in patients with chronic heart failure can also indirectly lead to neurological damage by promoting oxidative stress and inflammatory responses (Aldahl et al., 2017). In addition, disorders of lipid metabolism are recognized as major risk factors for many neurodegenerative diseases, including AD and PD. Disorders of lipid metabolism in the brain are closely associated with the pathological progression of these diseases (Wei et al., 2023) and may affect the levels of neurofilament chain proteins, but the exact mechanisms are not fully understood. And current studies do not provide sufficient evidence to establish a direct link between changes in lipid levels and serum potassium ion levels.

Elevated levels of sNfL, a biomarker of axonal damage, reflect the extent of neuronal axonal damage, which has been studied in a variety of neurological disorders. Elevated blood potassium may lead to elevated sNfL levels reflecting increased nerve cell damage through several mechanisms. First, it has been demonstrated that cerebellar granule neurons undergo rapid apoptosis in media with a high potassium environment (Harris et al., 2002). Elevated blood potassium levels can affect the excitability of neuronal cells, leading to neuronal hyperexcitability, and this hyperexcitability can trigger a series of biochemical responses that activate the MAPK and PI3K/Akt signaling pathways. The activated MAPK signaling pathway can affect cellular response to injury, including promotion of apoptosis (Teos et al., 2008; Yang et al., 2022). Whereas the PI3K/Akt signaling pathway plays a key role in regulating cell survival and apoptosis, its activation can lead to increased apoptosis (Devader et al., 2015), implying that neuronal overexcitability may trigger neuronal cell injury or death, which leads to elevated sNfL. Second, voltage-gated potassium channels in the nervous system are de-regulated by an anchor protein, which recruits other membrane proteins and ion channels to specific subcellular structural domains, and then stabilizes these subcellular structural domains through the interaction of the anchor protein with the submembrane spectrin cytoskeleton (Lazarov et al., 2018; Stevens and Rasband, 2022). The ionic concentration gradient inside and outside the nerve cell is altered in hyperkalemic states, and this alteration may affect cytoskeletal stability. Abnormalities in the cytoskeleton may lead to abnormal aggregation or degradation of neurofilament proteins, which are the major cytoskeletal proteins of neuronal axons, and their abnormal aggregation or degradation releases the light-chain subunit of neurofilament proteins (sNfL) into the cerebrospinal fluid and the blood circulation. sNfL serves as a biomarker for axon damage, and its elevated level reflects the extent of neural axon damage. Therefore, hyperkalemia may cause abnormal aggregation or degradation of neurofilament proteins by affecting cytoskeletal stability, which in turn causes increased release of sNfL, as has been demonstrated in a variety of neurological disorders. In addition, hyperkalemia may affect cell signaling pathways, such as activating microglia by activating N-methyl-D-aspartate (NMDA) receptors and increasing calcium inward flow, and may also activate microglia by increasing the release of inflammatory factors, such as tumor necrosis factor-alpha (TNF-alpha) and interleukin-6 (IL-6), which can directly stimulate microglia, prompting them to produce more inflammatory mediators, further exacerbating neuroinflammation and causing nerve damage (Bagheri-Yarmand et al., 2021; Liu et al., 2024; Merighi et al., 2022). Finally, hyperkalemia may trigger oxidative stress through several aspects, including affecting the process of intra- and extracellular ion exchange, leading to changes in cellular membrane potential and affecting normal cellular function and the normal operation of antioxidant defense mechanisms; the ionic changes triggered by hyperkalemia may also lead to mitochondrial dysfunction, affecting the process of oxidative phosphorylation, which may increase the production of reactive oxygen species (ROS); hyperkalemia can also affect the activity of antioxidant enzymes, such as superoxide dismutase (SOD) and catalase (CAT), which are the main defense mechanisms for scavenging ROS in the body. The accumulation of ROS exceeds the body’s antioxidant capacity, leading to oxidative stress (Al-Owais et al., 2015; Koong et al., 1993; Udensi and Tchounwou, 2017). These triggered oxidative stresses, which produce large amounts of ROS, can cause oxidative damage to nerve cells, which in turn leads to cell death. The combination of all these mechanisms can lead to nerve cell damage, which in turn leads to elevated sNfL levels.

In the realm of metal ions and neurological diseases, research exploring the link between potassium and axonal degeneration has been particularly active. This research focuses the potential role of serum potassium concentration, providing a valuable foundation for doctors to better manage and control these two biomarkers while minimizing the interference of confounding factors. The research also had several limitations; the cross-sectional report cannot determine a causal relationship between serum potassium levels and sNfL. Secondly, the research was limited to inhabitants in the United States and was not representative of the population as a whole, so the findings cannot be generalized to other populations with different health behaviors and hazard factors. In addition, the data of sNfL in NHANES was only collected from 2013 to 2014, which precluded further validation using NHANES data from the remaining time periods.

According to the current investigation, we found that among American adults, the greater serum potassium levels were linked to the higher level of sNfL, even following the adjustment for confounding variables.

## 5 Conclusion

The investigation found a significant positive correlation between serum potassium concentration and sNfL among adults in the United States. Higher serum potassium concentration was connected with the higher level of sNfL. Our findings suggest that elevated serum potassium levels may contribute to axonal degeneration. The potential utility of serum potassium concentration evaluation in identifying axonal degeneration underscores the clinical significance of our findings. Routine assessment of serum potassium may aid in early identification of individuals at risk for axonal degeneration-related diseases and guide personalized prevention strategies.

## Data Availability

The original contributions presented in this study are included in this article/supplementary material, further inquiries can be directed to the corresponding authors.
